# Controlling DNA Translocation Through Solid-state Nanopores

**DOI:** 10.1186/s11671-020-03308-x

**Published:** 2020-04-15

**Authors:** Zhishan Yuan, Youming Liu, Min Dai, Xin Yi, Chengyong Wang

**Affiliations:** School of Electro-mechanical Engineering, Guangdong University of Technology, Guangzhou, 510006 China

**Keywords:** Solid-state nanopores, DNA sequencing, Nanopores, DNA translocation

## Abstract

Compared with the status of bio-nanopores, there are still several challenges that need to be overcome before solid-state nanopores can be applied in commercial DNA sequencing. Low spatial and low temporal resolution are the two major challenges. Owing to restrictions on nanopore length and the solid-state nanopores’ surface properties, there is still room for improving the spatial resolution. Meanwhile, DNA translocation is too fast under an electrical force, which results in the acquisition of few valid data points. The temporal resolution of solid-state nanopores could thus be enhanced if the DNA translocation speed is well controlled. In this mini-review, we briefly summarize the methods of improving spatial resolution and concentrate on controllable methods to promote the resolution of nanopore detection. In addition, we provide a perspective on the development of DNA sequencing by nanopores.

## Introduction

In recent decades, much progress has been made in applying DNA sequencing to read the sequences of bases in the genome [1, 2]. To develop personalized medicines, researchers have been seeking a faster and cheaper DNA sequencing method, where target drugs and medical treatments can be applied to an individual specifically [3, 4]. Because nanopore technology was used in DNA detection [5], it was considered to be an effective method for next-generation sequencing [6, 7]. Nanopore technology is a promising platform to identify highly sensitive, single-molecule detectors for DNA [5] or RNA [8]. In the basic detection scheme, an electrochemical chamber is separated into two reservoirs (cis- and trans-compartments) by a thin membrane with a nanopore [9], which connects the conductive solution and the analyte in the electrochemical chamber. By applying a voltage across the membrane, electrolyte ions flow through the solid-state nanopore and form a pore current, which is measured using a patch clamp set-up with associated ultra-sensitive electronics. When a molecule or molecular complex passes through the nanopore, the analyte can exclude some ions from the volume defined by the nanopore, which can be detected by monitoring brief changes in the current. From both residence time (dwell time) within the nanopore and the current amplitude signature, information about the molecules can be obtained. The spatial resolution of nanopore sequencing is determined by the dimensions of the nanopore, which suggests that it can be used as a single sensor for small molecular objects resulting in a detectable current signature. Moreover, nanopore sensors are easy to integrate into highly portable lab-on-a-chip devices and miniaturize [10].

Significant progress has been made in DNA sequencing by nanopores, such as solid-state nanopores [2, 11] and protein nanopores [2, 7]. DNA sequencing by protein nanopores has been achieved [7]. However, protein nanopore systems have limits for studying biological molecules. There are fewer constraints with solid state nanopores as compared to biological/protein nanopores. These difficulties may be overcome by solid-state nanopores. Compared with protein nanopores, they are functional over wider ranges of temperatures, voltages, and solvent conditions and can be tuned in diameter with sub-nanometer precision. They are promising for application in next-generation technology for DNA sequencing.

Many solid-state nanopores of different materials and structures have been made for DNA sensors. However, DNA sequencing is not achieved by solid-state nanopores. For solid-state nanopore sensors, there is a need to overcome two major obstacles, regarding their spatial resolution and temporal resolution, before their commercial application to DNA sequencing. The difficulty regarding spatial resolution is that the solid-state nanopore can distinguish the tiny spacing between two neighboring nucleotides in order to achieve single-base resolution. The obstacle of temporal resolution is that DNA translocation is too fast under an electrical force, which results in the acquisition of very few valid data points by existing patch clamps or other signal acquisition systems. This mini-review presents an overview of the various methods to improve the spatial resolution and temporal resolution of solid-state nanopore DNA detection. This mini-review also focuses more on methods of slowing down DNA translocation through solid-state nanopores.

## Spatial Resolution

In 2001, silicon nitride solid-state nanopores were first reported by Li et al. [12]. Various solid-state nanopores have been demonstrated for DNA molecular detection, such as silicon oxide [13], silicon [14], Al_2_O_3_ [15], and HfO_2_ [16]. While solid-state nanopores may ultimately be robust to chemical and mechanical conditions, they have some limitations, such as low spatial resolution. Owing to the thickness of materials, dozens of bases can pass solid-state nanopores at a time. At present, the thinnest silicon nitride nanopore is 3 nm, which still does not distinguish the four kinds of base [17].

Interestingly, the thickness of a single layer of two-dimensional (2D) material is approximately 3.0–11.0 Å, which is comparable to the spacing between two neighboring nucleotides along ssDNA (3.2–5.2 Å) [18]. Two-dimensional membranes, such as graphene (3.4 Å [19]), MoS_2_ (6.5 Å [18]), WS_2_ (7 Å [20]), and h-BN (11 Å [21]), have been demonstrated to detect DNA translocations [21–23] because of their high signal-to-noise ratio and spatial resolution. It is clear from their spatial resolution that those materials can be used for DNA detection. Besides, reproducible growth techniques and large-scale transfer procedures make it possible to fabricate sub-nanometer pores in 2D membranes on a large scale.

Graphene is an atomically thin sheet of carbon atoms arranged into a two-dimensional honeycomb lattice [24]. Researchers have demonstrated that single DNA molecules in solution can be detected and characterized with graphene nanopores [22, 25]. However, there are strong hydrophobic interactions between DNA nucleotides and graphene, and the DNA will severely clog and stick to graphene nanopores, which would markedly impact on the translocation speed [26]. Modified with the hydrophilic groups [26] or coated, a hydrophilic material [25] on graphene could improve the hydrophilicity of graphene and avoid DNA sticking to its surface. Unfortunately, either the modification with hydrophilic groups or the coating with hydrophilic materials will increase the thickness of the suspended film, leading to the increase in the thickness of the nanopores, thus decreasing the spatial resolution of graphene.

Layered transition metal dichalcogenide is another 2D material, which includes MoS_2_ [18, 27] and WS_2_ [20]. High signal-to-noise ratio (SNR > 10) and five-fold enhancement of the ionic current signal were detected when DNA translocated through the MoS_2_ nanopore membrane [18]. Meanwhile, MoS_2_ is hydrophilic, so no special surface treatment is needed to avoid hydrophobic interaction between DNA and its surface. The other material, WS_2_ has a direct band gap of 2.1 eV [28], and its photoluminescence (PL) emission is stronger than that in the well-studied MoS_2_ [29]. Danda et al. [20] fabricated a WS_2_ nanopore and demonstrated the achievement of atomically controlled nanopore size using short light pulses, which may have positive effects on solid-state nanopores for DNA detection.

Besides, Liu et al. [21] reported the first experiment of DNA translocation through h-BN nanopores. Similar to graphene, h-BN has poor hydrophilicity, which will cause DNA to block nanopores. Subsequently, Zhou et al. [23] successfully improved the hydrophilicity of h-BN nanopores by taking advantage of the antioxidation and integrity of h-BN material after UV-ozone treatment. The insulating nature of h-BN may exhibit more remarkable durability and insulating properties in high-ionic-strength solution than in graphene. It is a competitive candidate to achieve single-base resolution on super-thin nanopore structures.

The use of two-dimensional materials can potentially increase the spatial resolution of the device to achieve single-nucleotide resolution. Although DNA detection experiments for several two-dimensional materials have been reported, no one has reported the achievement of solid-state nanopore DNA sequencing. The temporal resolution of nanopore sequencing is also a challenge.

## Temporal Resolution

**Fig. 1:**
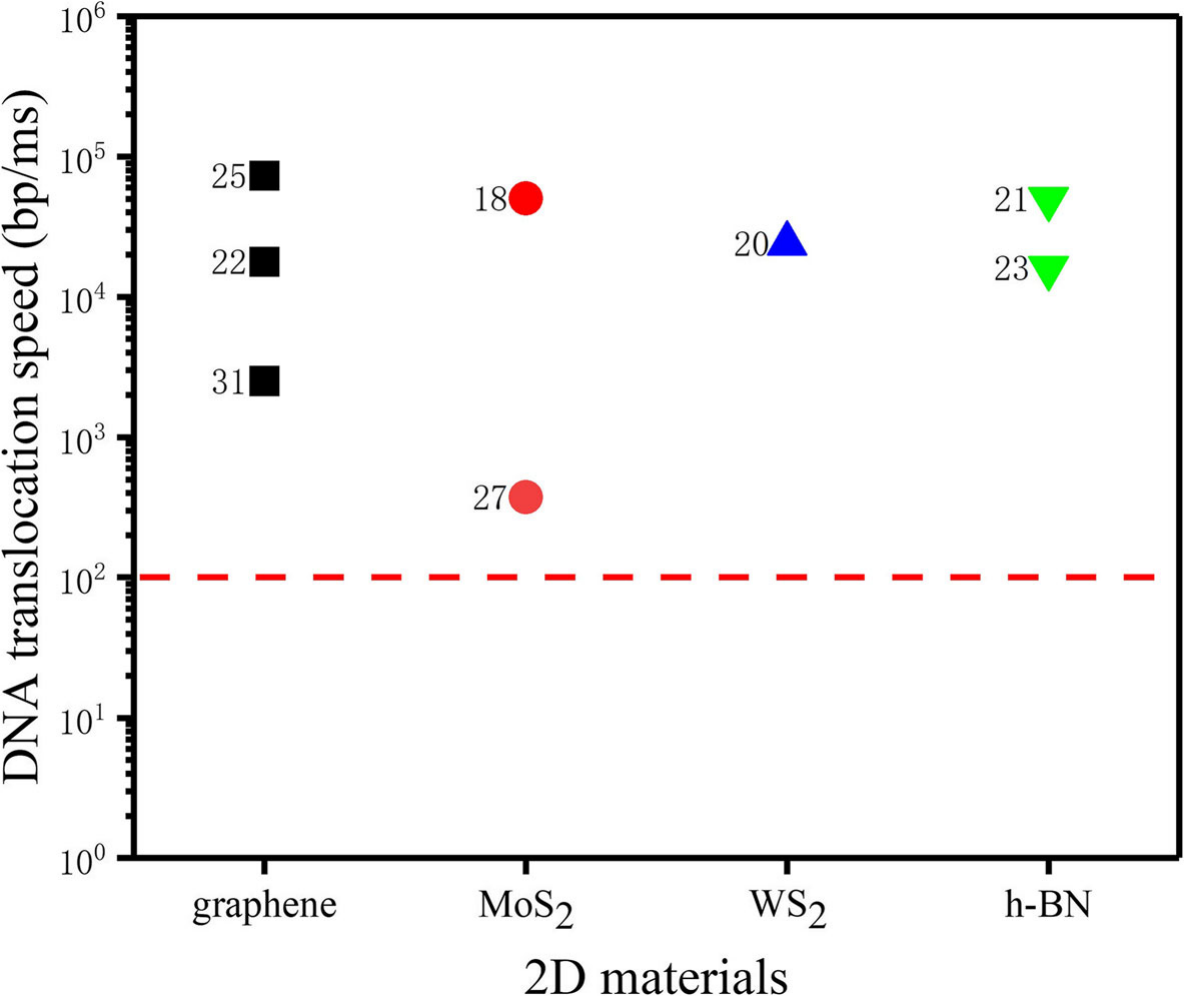
DNA translocation speed of 2D solid-state nanopore [18, 20–23, 25, 27, 31]. The dotted red line indicates a DNA translocation rate of 100 bp/ms

The DNA translocation speed through a solid-state nanopore is very fast, at up to 0.01–1 μs/base [30], leading to very little effective data collected by commercial patch clamps. As such, it is not possible to distinguish every base based on the blockade current signal. The DNA translocation speeds of solid-state nanopores of 2D materials, such as graphene, MoS_2_, WS_2_, and h-BN, are shown in Fig. 1. Ideally, the DNA translocation speed in a nanopore should be 1–100 bp/ms to enable satisfactory recording of the signal from each nucleotide [32].

Against this background, slowing down the translocation speed of DNA is an important objective pursued by many researchers. Various methods have been developed to slow down DNA translocation to improve the temporal resolution of solid-state nanopore detection. The usual method is to change the influence of experimental factors such as temperature [33], electrolyte viscosity [27], driving voltage [34], ion concentration [35], and surface charge density of a nanopore [36] by changing the DNA translocation through it. Wanunu et al. [33] concentrated on slowing down dsDNA translocation through solid-state nanopores by changing the temperature, voltage, and DNA length. Moreover, Feng et al. [27] showed that a viscosity gradient system, based on room-temperature ionic liquids, can be used to control the dynamics of DNA translocation through MoS_2_ nanopores, and demonstrated that the high viscosity of room-temperature ionic liquids provides optimal single-nucleotide translocation speeds of 373 bp/ms.

Although many approaches have the potential to reduce DNA translocation speed and facilitate ionic current detection, they still cannot meet the requirements for DNA sequencing. Therefore, it is necessary to develop more radical methods to control the passage of DNA through nanopores. Here, we discuss the methods of controlling nanopore structure and quantitative DNA movement to improve the temporal resolution.

### Two Nanopores System

**Fig. 2:**
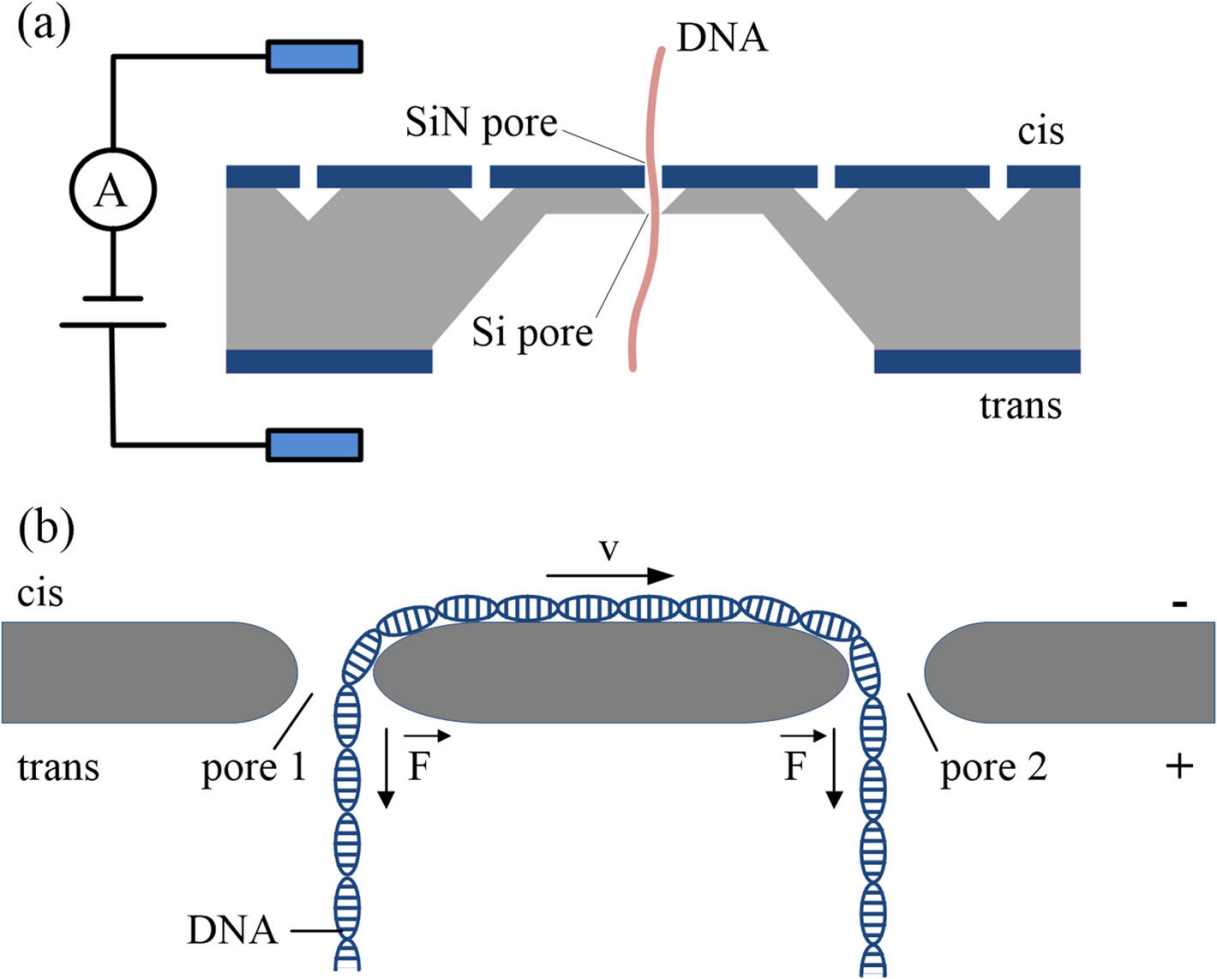
**a** Schematic of two-layer nanopores system used for nanopore DNA detection [37]. **b** Schematic of double-nanopore system used for nanopore DNA detection [38]

Researchers have used two-nanopore systems to manipulate DNA molecule translocations, which can controllably detect the same molecule many times. Two stacked nanopores, separated by a micrometer-sized cavity compartment [37] (Fig. 2a), can trap DNA molecules for a certain amount of time and controllably release them. The dynamics of DNA molecules can be deduced from signals of the two pores by correlation analysis, which provides direct electrical proof for translocation. In addition, because of the entropy barriers of the two-layer nanopore system, the Brownian motion of DNA molecules can be confined, which can improve DNA sequencing accuracy with nanopores. Compared with single-molecule sensing techniques, DNA molecules can be measured multiple times in two-layer nanopore systems by serially arranging multiple pores instead of passing them back and forth through a single pore [39].

Double-nanopore systems provide another method to control molecular transport and efficiently bridge molecules between two pores; they are a label-free mechanistic approach for DNA manipulation [40]. In a double-nanopore system, there are two independently addressable and adjacent nanopores within the same solid-state membrane. During the electrophoretically driven passage of a DNA molecule through one of the nanopores, a single DNA molecule can be captured into both pores, leading to a “tug-of-war” between the two nanopores [38] (Fig. 2b). Therefore, forces are applied to the different ends of a DNA molecule, which slows down and even fully arrests its motion. The double-nanopore system opens up a new path to the mechanical trapping of DNA in solid-state nanopores, and it is a promising technique to measure a wide range of biomolecules with the advantages of being label-free, and having a high signal-to-noise ratio and low cost. It can efficiently confine and trap the DNA molecules to slow down DNA translocation and can also be used to study the physics of this nanoscale tug-of-war on DNA [41].

### Optical Trap Nanopore

**Fig. 3:**
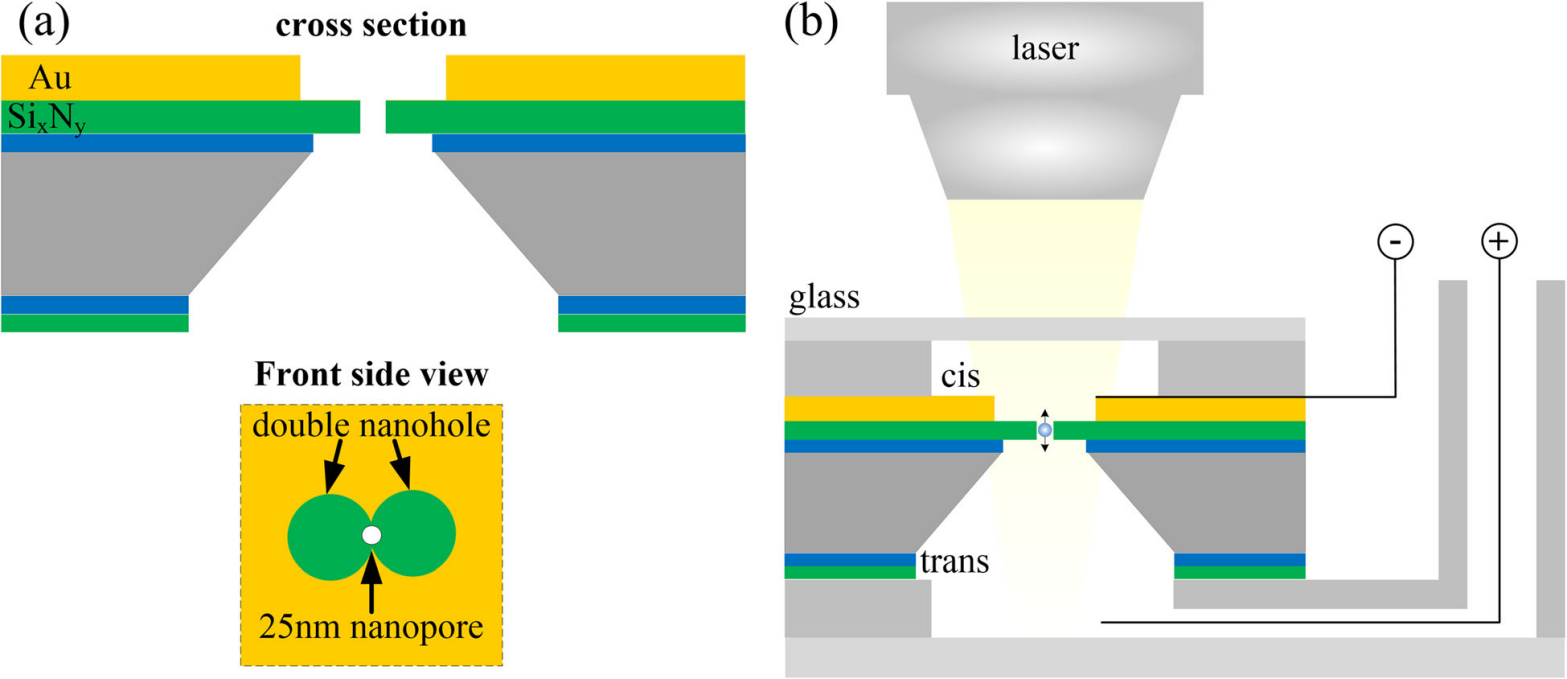
**a** Schematic of the Optical trap nanopore chip [47]. **b** Schematic of the self-induced back-action system

Optical trap nanopores allow optical tweezers to trap particles of less than tens of nanometers in size. It makes possible the optical trapping of proteins [42], DNA fragments [43], and other biomolecules [44], as well as small viruses [45]. The basic theory behind optical trap nanopores is a self-induced back-action optical trap [46]. The laser beam focused on the region of the nanopore array will form a high-power density local light field at the edge of the metal layer in the hole. As a particle moves between the local light field, it can cause a large change in the local optical transmission, which will in turn produce a large optical and dielectric force on the particle. A double nanohole structure is used to break the size bottleneck of captured particles. Muhammad et al. [47] demonstrated the potential use of optical trap nanopores with 20-nm silica and Au nanoparticles. The dumbbell shape of the double nanohole was milled into the Au film, and a 25-nm nanopore was drilled through the suspended Si_x_N_y_ membrane in the middle of the double nanohole, as shown in Fig. 3a. Electrophoretic force driving nanoparticles through the nanopore, when passing through the edge of the hole, the self-induced back action plasmonic force existing between the tips of the double nanohole opposed the electrophoretic force, reducing the speed of the nanoparticles. The results showed that the optical trapping extended the electrophoretic translocation time by four orders of magnitude. Kim et al. [43] realized the optical capture of plasmid DNA and lambda DNA by using the nanoplasmonic structures of single nanopores. The technology has potential applications for DNA detection and multiple local light fields can be set up to realize parallel detection. However, high-frequency ion current oscillation may affect the current detection results of DNA. This may be due to the competition between electrophoretic and self-induced back-action forces causing the nanoparticles to float up and down through the mouth of the nanopore.

### Optical tweezers

**Fig. 4:**
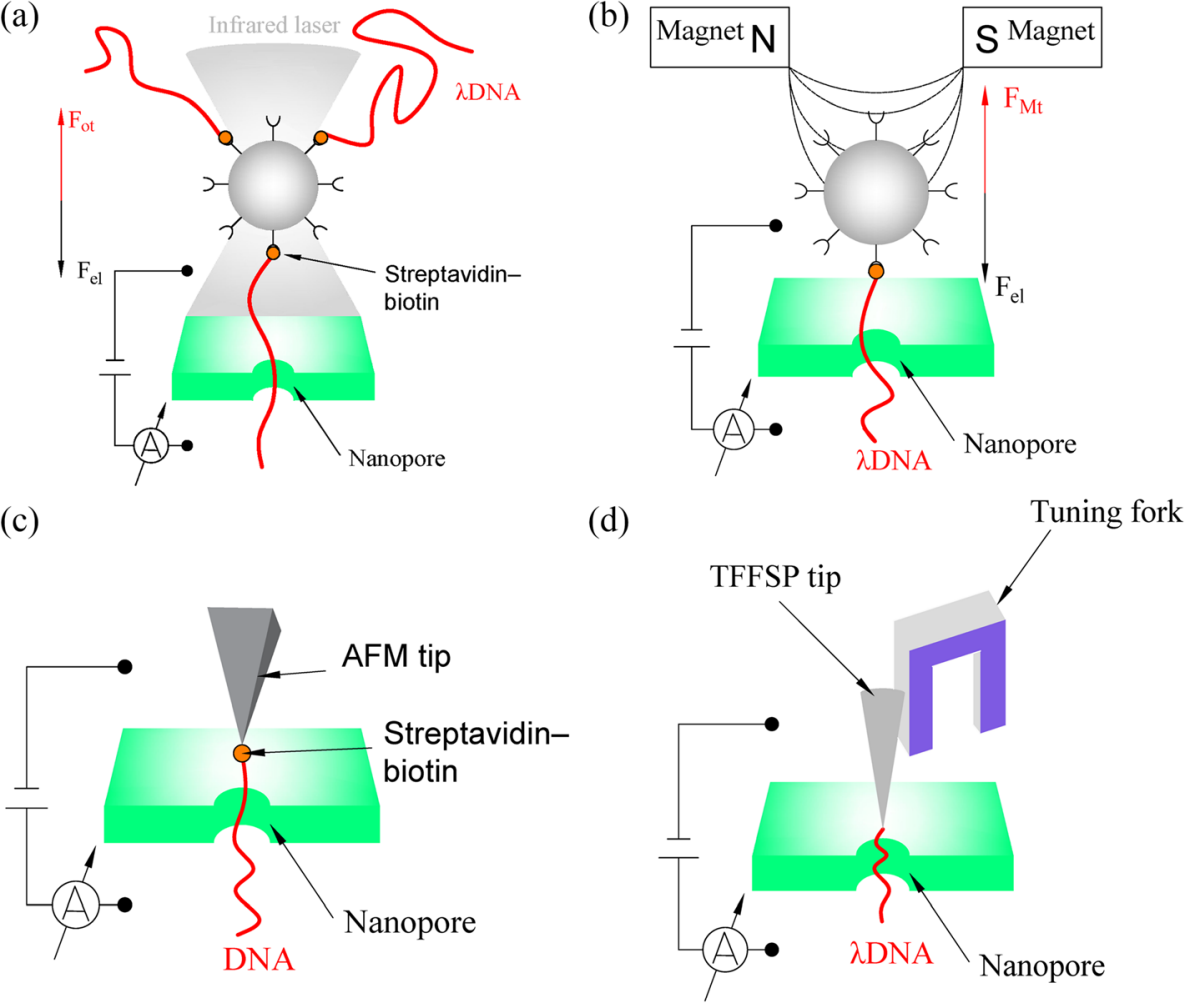
**a** Schematic of optical tweezers used for nanopore DNA detection [48]. When the optical tweezers system is in equilibrium, the optical force (F_ot_) is equal to the electric field force (F_el_). **b** Schematic of magnetic tweezers used for nanopore DNA detection [49]. When the magnetic tweezers system is in equilibrium, the magnetic force (F_Mt_) is equal to the electric field force (F_el_). **c** Schematic of AFM used for nanopore DNA detection [50]. **d** Schematic of TFFS used for nanopore DNA detection [51]

Optical tweezers can be used to control molecular translocation through nanopores and have been commonly used in recent years. In 2006, Keyser et al. [48] first demonstrated a molecular tug-of-war between electrophoretic and mechanical forces by applying optical tweezers to control DNA translocation through SiN_x_ nanopores. This system acts as a simple Hooke spring, and the tension force on the bead can be calculated based on Hooke’s law: *F*_ot_*= −k*_trap_*Z*, where *F*_ot_ is the optical force, *k*_trap_ is the trap stiffness along the displacement direction, and *Z* is the linear deformation of the bead [48]. The optical tweezers method, which traps a DNA-tethered polystyrene bead in the crossover of a focused laser beam, can manipulate the DNA-tethered polystyrene bead in three dimensions and has a pico-newton range of force sensitivity, as shown in Fig. 4a. To trap the translocating DNA inside the nanopore, the tension force was tuned to balance the electric field force (*F*_el_) on the DNA. Therefore, the tension can be used to both reduce the speed of DNA translocation and pull the DNA molecule out of a nanopore [52]. This system permits simultaneous spatial sampling and high-resolution force measurements of nucleic acids and proteins, which has achieved significant progress in DNA sequencing. However, the optical tweezers technique suffers from several fundamental difficulties. First, it is difficult to scale up to a large number of nanopores. Second, the heating caused by the laser in optical tweezers strongly impacts on the ionic current through a nanopore and the noise level, requiring the optically trapped bead to be several micrometers away from the nanopore [53].

### Magnetic Tweezers

Magnetic tweezers provide another way of controlling DNA translocation by tension force, and it has been demonstrated that the magnetic tweezers technique is effective in slowing DNA translocation [49]. In this system, as shown in Fig. 4b, DNA molecules can be attached to a micrometer-sized magnetic bead using strong gold–thiol [54] or streptavidin-biotin [49] interaction. Then, the free end of the DNA can be captured in the nanopore by an applied electric field. Subsequently, two magnets with a small gap can be used to create a gradient of magnetic field. This technique can balance the electrical force on the trapped DNA to reduce translocation speeds and even reverse electrophoresis. Compared with optical tweezers, magnetic tweezers are a promising candidate for massively parallel force spectroscopy. In this system, hundreds of beads and thus DNA molecules can be simultaneously controlled within hundreds of nanopores, which is easily scalable to many addressable nanopores. This can speed up the analytical process by orders of magnitude. However, compared with optical tweezers, one obvious disadvantage of the magnetic tweezers approach is the lack of three-dimensional control of the molecules [55].

### Force Sensing Probe

Although significant progress has been made in controlling DNA translocation speed by optical tweezers and magnetic tweezers, the bead-trapping methods have a problem with Brownian motion that makes it difficult to control the motion of the bead with less than 10-nm resolution [51]. To overcome this, AFM has been used to control the speed of DNA translocation [50], and it can also simultaneously measure the force and the blockade current. In a study using this system, as shown in Fig. 4c, DNA was tethered to the tip of an AFM probe, and then it was clamped into the probe holder. By controlling the motion of the probe, the DNA translocation could be controlled to reduce its speed and even reverse electrophoresis. Furthermore, by retracting the tip to a height above the surface corresponding to the length of the molecule, the measurements can be repeated. With AFM assistance, DNA detection has advanced in practice and theory because the detection resolution can be greatly improved by the combined signals of blockade current and AFM force measurements [56]. However, there is still an obstacle in the application of nanopore techniques in DNA detection, namely, the intermittent occurrence of regular fluctuations in the force (and the current) every 0.35–0.72 nm when a DNA molecule slides in a relatively frictionless manner through a nanopore. These fluctuations are attributed to individual nucleotides translating through the nanopore in a turnstile-like motion [50].

Studies have demonstrated that a tuning fork, which can be used as a force detecting sensor, can control DNA to pass through a nanopore at a sub-nanometer rate [51, 57]. In a study using this integrated apparatus, as shown in Fig. 4d, a DNA molecule was attached to a probe tip that was glued to one prong of a tuning fork. A nano-positioning system, which possesses sub-nanometer accuracy, was used to hold the tuning fork [51]. The position of the probe tip can be sensed by the tuning fork-based feedback force sensor and controlled by manipulating the nanometer positioning system. This movement speed is 10 times slower than that of DNA manipulated by optical tweezers and 1000 times slower than DNA passing freely through solid-state nanopores [57]. Compared with conventional AFM, a tuning fork can provide faster scan motion and possess high force sensitivity when immersed in the liquid. By incorporating TFFS with a nanopore, the ionic current through a nanopore, tip position, and tip vibrational amplitude can be simultaneously measured during the passage of a DNA molecule through the nanopore.

### Si Probe

**Fig. 5:**
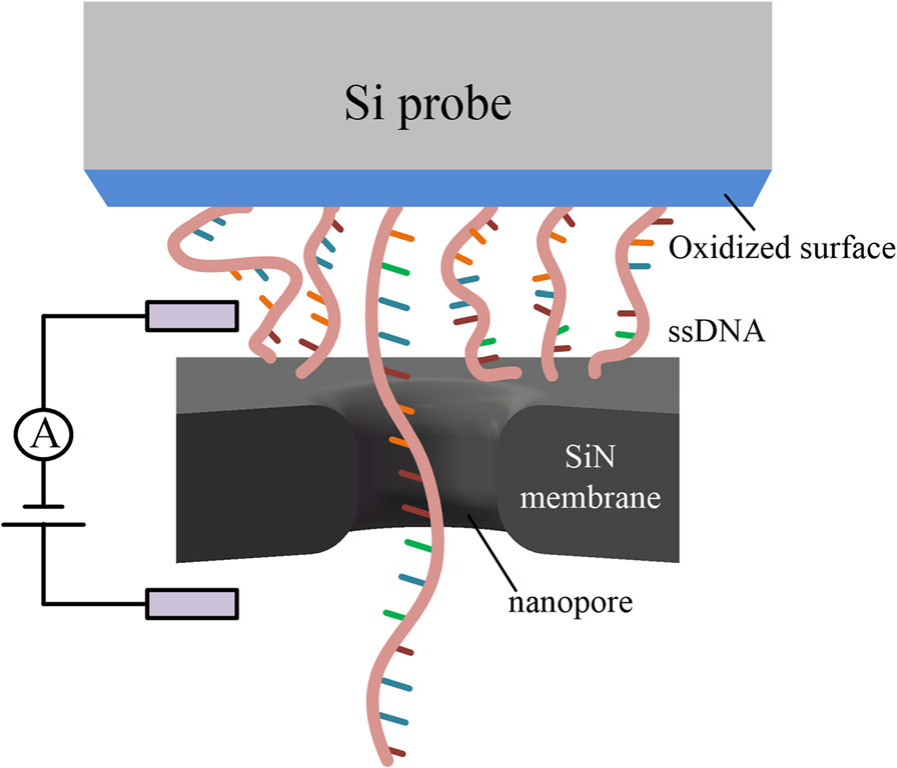
Schematic of the DNA-immobilized Si probe system used for nanopore DNA detection [32]

All of the methods above, namely, magnetic tweezers, optical tweezers, AFM, and TFFS, need to scan the nanopore position. They have to locate the nanopore within the effective length of the DNA to make sure that the DNA can pass through the nanopore. Nanopore addressing is an important part of these methods but is difficult [32] connected DNA to a large surface of a silicon probe (Fig. 5), larger than the area of the chip, which meant that they could easily insert the immobilized DNA into a nanopore without scanning for the location of the nanopore in the membrane. The feasibility of using a DNA-immobilized Si probe and position controller to control the movement of DNA into and out of nanopores has been demonstrated. The difficulty of this method is that the Si probe is immersed in the solution and connected to the DNA by the peptide coupling. The density of DNA on the probe’s surface is difficult to control, so multiple DNA passing through a nanopore at the same time is likely to occur, affecting the detection current.

Optical tweezers, magnetic tweezers, atomic force microscopy (AFM), and tuning fork-based force sensing (TFFS) can detect the actual forces and position of the molecule in the nanopore, which is promising to control DNA passage through nanopores with appropriate speed. The difficulty of addressing nanopores is avoided by using a Si probe. In addition, the use of a two-nanopore system is a feasible method to control and slow down DNA passage through nanopores. In addition, an optical trap nanopore has potential for DNA detection in the future. Here, we summarized the DNA translocation speed of a solid-state nanopore integrated with some DNA control methods, such as a two-nanopore system, optical trap nanopore, optical tweezers, magnetic tweezers, AFM, TFFS, and Si probe (Table 1).

**Table 1:** DNA translocation speed with some DNA control method

Control method	Advantages	Disadvantages	DNA translocation speed (bp/ms)
Two nanopores system	Continuous collection of signals, high signal-to-noise ratio, low cost	Difficulty of mass-controlled manufacturing	~ 1429 [37]
Optical trap nanopore	Label-free, parallel detection	Difficulty of mass-controlled manufacturing	—
Optical tweezers	Three-dimensional control	Cannot realize parallel detection, the heating impact	~ 150 [52]
Magnetic tweezers	Massively parallel detection	Force hysteresis	~ 10 [49]
AFM	Easy to control with less than 10 nm resolution	Effect of probe on nanopores	~ 0.06 [50]
TFFS	Faster scan motion, high force sensitivity	Effect of probe on nanopores	~ 1 [57] ~ 10 [51]
Si probe	No need for nanopores addressing	Adjacent DNA affects accuracy	~ 0.1 [32]

## Conclusion

Monolayer 2D materials, such as graphene, MoS_2_, WS_2_, and h-BN, are possibly the thinnest achievable materials as they are as thick as the spacing between the nucleotides. Compared with traditional solid-state-nanopore membranes, monolayer 2D membranes are ideal for nanopore devices as they exhibit high ionic current signal-to-noise ratio and relatively large sensing regions. They are potentially eligible to realize DNA sequencing by combining with optical tweezers, magnetic tweezers, AFM, TFFS, Si probe, two-nanopore system, or optical trap nanopore. However, with these techniques, several challenges have arisen, which need to be resolved before the commercialization of nanopore DNA sequencing. The first of these occurs when the beads or probe tips are close to a nanopore, when it is more difficult to discriminate DNA nucleotides with ionic current signals. Molecular handles or other longer molecules should be used to add the length of a DNA strand that can offset the effect on current signal brought about by beads or tips. Second, nanopore arrays should be used to realize high-throughput and parallel detection, but the technology of parallel detection is currently not mature enough. Third, according to present fabrication methods, it is difficult to fabricate a two-nanopore system and optical trap nanopore system with high accuracy and reproducibility, which is very significant for nanopore DNA detection. A helium ion beam may be the key technology to solve this problem [11, 58]. Thus, we expect that DNA nanopore sequencing will continue to be a research focus and can be integrated with more new ideas and innovative approaches to realize low error rates, rapid and high parallel recording, and long read lengths of up to 100 kilobases.

## Data Availability

All data generated or analyzed during this study are included in this published article.

## Abbreviations

2D: Two-dimension
3D: Three-dimensional materials
TMDs: Layered transition metal dichalcogenide
SNR: Signal-to-noise ratio
PL: Photoluminescence
SIBA: Self-induced back-action
AFM: Atomic force microscopy
TFFS: Tuning fork based-force sensing
